# Risk-Based Learning Games Improve Long-Term Retention of Information among School Pupils

**DOI:** 10.1371/journal.pone.0103640

**Published:** 2014-07-29

**Authors:** Ian M. Devonshire, Jenny Davis, Sophie Fairweather, Lauren Highfield, Chandni Thaker, Ashleigh Walsh, Rachel Wilson, Gareth J. Hathway

**Affiliations:** Laboratory of Developmental Nociception, School of Life Sciences, Nottingham University Medical School, Queen's Medical Centre, Nottingham, United Kingdom; Georgetown University Medical Center, United States of America

## Abstract

Risk heightens motivation and, if used appropriately, may have the potential to improve engagement in the classroom. We have developed a risk-based learning game for school pupils in order to test whether such learning games can improve later recall of information. The study was performed during a series of public engagement workshops delivered by undergraduate students. Undergraduate neuroscience students delivered 90-minute science workshops to 9–10 year old school pupils (n = 448) that were divided into ‘Risk’, ‘No risk’ and ‘Control’ classes. ‘Risk’ classes received periodic multiple-choice questions (MCQs) during the workshops which required small teams of pupils to assign tokens to the answer(s) they believed to be correct. Tokens assigned to the correct answer were returned to the group and an equal number given back as a prize; tokens assigned to incorrect answers were lost. Participation was incentivised by the promise of a brain-related prize to the team with the most tokens at the end of the workshop. ‘No risk’ classes received MCQs without the risk component whilst the ‘Control’ classes received no MCQs. When presented with a neuroscience quiz based on workshop content at the end of the workshop, pupils in the ‘Risk’ classes exhibited significantly greater recall of information one week later. Quiz scores were higher than scores from the day of the workshop which suggested pupils may have discussed the workshop content outside of the classroom, thereby increasing knowledge over and above what was learned during the workshop. This is supported by feedback from pupils in ‘Risk’ classes which indicated that ‘Risk’ workshops were more interesting than ‘No risk’ and ‘Control’ workshops. These data suggest that there is a role for risk in the classroom but further investigations are required to elucidate the causal mechanisms of improved retention of information.

## Introduction

High levels of engagement and attention facilitate learning in the classroom and, in an effort to improve academic performance, educators are constantly developing new approaches and strategies to better engage young learners. One such approach is the use of learning games in the hope that the enjoyment and intrinsic motivation so often associated with entertainment games will carry over into their educational equivalent [1]. Another major benefit of learning games is that they encourage participation by reducing the negative effects of failure on self-esteem that other teaching styles, such as direct questioning, can cause. The research field that has built up around this approach is known as game-based learning [2], [3], [4], [5]. Attempts at emulating the popularity of entertainment games in the classroom however, especially using personal or tablet computers, has had mixed success [1], [6] and the translation of skills acquired in a game to real world contexts is often poor [7], [8]. One possible reason for inconsistent results in this field is that the critical factor(s) that make entertainment games so compelling is hotly debated and/or when identified, is poorly integrated into the educational game [2]. The inclusion of multiple experimental conditions to enable comparison between different teaching strategies instead of purely pre/post testing schedules (i.e. between learning games and more traditional teaching styles) does not happen commonly enough in the game-based learning field and may help identify subtle benefits of educational games [8]. However, it has recently been proposed that the inclusion of an element of risk into computer or classroom-based learning games might be crucial in engaging learners and improving retention of information learned during the game [9].

Risk heightens motivation [10] and moderate risk could, therefore, be initially used to engage otherwise disinterested pupils. Risk-taking can be defined as “the participation in behaviour which involves potential negative consequences (or loss) balanced in some way by perceived positive consequences (or gain)” [11]. Adolescents are more tolerant of ambiguously risky situations than adults [12] and risk-taking behaviour peaks at an age when brain networks responsible for socio-emotional behaviour (e.g. amygdala, ventral striatum, superior temporal sulcus) develop more rapidly than those necessary for cognitive control (such as the lateral prefrontal and parietal cortex) [13], [14], allowing stimulus-driven behaviour to overrule goal-driven behaviour. Indeed, this difference between the developmental rate of brain regions is believed to allow organisms to better take advantage of learning opportunities in their environment [12]. Thus, childhood could be an opportune time to utilise the benefits of risk-based learning strategies. Eventually, as the cognitive control network catches up and integration between brain areas improves – especially in the frontal cortex [15], [16] – perceptions of reward and risk are sharpened, impulsive behaviour is inhibited and the child slowly learns to balance short-term gains with long-term consequences, a vital skill for adulthood [17], [18], [19], [20].

We developed a risk-based learning game for pupils aged 9–10 years old (UK Year 5) that was incorporated into school science workshops delivered by undergraduate (UG) neuroscience students; the project was referred to as BrainLab [21]. Pupils played the game in small, competing groups and, therefore, the game can be classified as a form of team-based learning [22], an approach primarily used to develop pupils' interpersonal skills and problem-solving abilities (though these were attributes not specifically assessed in the current study). In our learning game, teams were required to work together to find the correct answer to periodic multiple-choice questions (MCQs) which included an element of risk by requiring each team to bet tokens on different answers.

The manner in which tokens are used in our learning game can be interpreted as the level of confidence pupils have in different answers. There are a number of methodologies used within educational psychology that record learner confidence to evaluate different attributes of the learning process and, therefore, share similarities with our approach. These methodologies include calibration, judgements of learning (JOLs) and confidence-based marking (CBM). Calibration compares confidence with accuracy [23], [24], JOLs examine the learner's confidence that information will be remembered [25], whilst CBM is strictly a form of assessment in which the level of confidence in an answer results in a respective penalisation or boost to the final score according to whether the learner is right or wrong [26], [27]. Our learning game is most similar to CBM and could be viewed as a team-based, tokenised version of the traditional CBM paradigm. However, it differs fundamentally from CBM in that we are not using the game for assessment purposes. By playing the game at key points throughout the workshop, in-between specific workshop activities, with each game question related to the workshop activity that immediately preceded it, the main purpose of the risk-based game is to enhance concentration and engagement during the activities and, therefore, increase the overall information learned. In this respect, our approach also differs from the majority of learning games which incorporate the information to be learned into the game itself and also tend to be computer-based. Thus, although participants in our workshops very much perceived the series of risk-based MCQs as a game, our approach could be considered instead as a novel teaching paradigm that borrows ideas from assessment strategies and learning games. Nevertheless, the approach will continue to be referred to as a learning game; its merits will be evaluated primarily by examining pupils' short- and long-term retention of workshop content, as well as comparing these data to a range of self-reported feedback responses.

## Methods

### Participants, recruitment and ethical consent

Ethical clearance for the project was obtained from Nottingham University's Medical School Ethics Committee (O12072012-BMS). A total of 448 pupils (229 boys, 219 girls) divided between 18 classes from 13 schools were recruited for the project (9–10 years old; Year 5). Average class size was 24.9 pupils (SD = 4.6; range = 16 to 35) with an average male∶female ratio of 51∶49 (range = 38∶62 to 64∶36). Recruitment was performed by the University of Nottingham's Widening Participation (WP) team and IntoUniversity, a national charity that provides university experience and academic support for school pupils in disadvantaged areas. Both the WP team and IntoUniversity utilised existing school networks within the Nottingham area to fit in with the wider context of the University of Nottingham WP initiative to raise awareness of higher education among disadvantaged schools in the local area. During recruitment, head teachers were provided with an information sheet that described the project and its aims together with a list of possible workshop dates. If the school wished to participate, a registration form was completed which allowed the school to provide contact details for an appropriate point-of-contact (as this is not always the head teacher), available workshop dates and the opportunity to highlight any special requirements or make specific comments. Ethical consent forms were sent to all schools that had returned a registration form; an individual consent form was sent to each head teacher and all parents were sent an opt-out form along with an information sheet that described the project. Therefore, head teachers provided written informed consent for the project as a whole whilst parents/caregivers were given the option to opt out (note that no parents/caregivers chose to opt out of the current study). Each aspect of the consent procedure was approved by Nottingham University's Medical School Ethics Committee.

The workshops were delivered as part of an UG science communication course which has been described previously albeit briefly [21]; the course represents a new model of public engagement as it combines UG teaching, public engagement and research. To enable replication of the model, full methodological details such as how workshop ideas were developed are provided in the current article. Six final (3^rd^) year undergraduate students were recruited to the project in an annual preview session in which each academic offering a project provides a brief description of their project to all final year undergraduate students. All academics offering projects receive teaching credit for supervision provided. Every student in their final year must choose either a research project (normally laboratory-based) or a library project. Following a preview session of all available projects students rank their top three choices. All students involved in our project ranked the BrainLab project in first place.

### Experimental Design

The independent variable in the design was workshop delivery style, with three conditions that differed in the amount and style of information reinforcement: no reinforcement (‘Control’), five equally-spaced multiple-choice questions (MCQs) answered in small teams that re-capped what had just been taught (‘No risk’) or the same MCQs in which teams could compete with one another by betting tokens on the correct answer (‘Risk’). The inclusion of the ‘No risk’ condition controlled for the novelty of the neuroscience workshop. Teams betting tokens on the correct answer received double the number of tokens back; to provide incentive, it was announced that the team with the most tokens at the end of the game would win small brain-related prizes, though these were not revealed until the end of the workshop (pencils and brain-shaped erasers, donated by The Dana Foundation). School classes were divided such that there were six classes in each experimental condition; all classes were treated as independent. This enabled each of the six undergraduate students to deliver one workshop in each of the three conditions and the data pooled. The order that each student delivered a workshop from each condition was counterbalanced to avoid order effects (e.g. increasing confidence and proficiency of the UG in their delivery). The primary dependent variable was the score from a neuroscience quiz (out of eight) which was completed immediately after each workshop (day 0) and again one week later (day 7). Secondary dependent variables were obtained from the results of an evaluation completed along with the neuroscience quiz immediately after the workshop.

### The risk-based learning game

Each class was divided into teams of 4–6 pupils that at five times during the workshop came together to answer an MCQ. One UG helper took charge of each team during the game. Each team were given 20 tokens which they could risk on any of the answers to the question, with the caveat that 10 tokens were always kept back until the last question (ensuring no team could lose all their tokens – and engagement – before the end of the game). For instance, the teams could risk the maximum number of tokens on a single question but lost them all if they got the question wrong, or else spread their tokens across a number of answers. The team received double the number of tokens that had been risked on the correct answer. All other tokens risked on incorrect answers were taken away. The question and five possible answers was read out by the lead UG before pupils made their choices. For example, one question in a workshop entitled ‘Super Synapses’ was ‘What is a chemical message kept inside before it enters the synapse?’, with possible answers A) Vehicle, B) Vertebra, C) Vesicle, or D) Veegram. Plastic pots were provided to represent each answer into which pupils could place their tokens. UG helpers kept track of scores and helped to make sure all members of the pupil team were involved in the decision process. In some instances, pupils decided to divide the tokens between each pupil and allow everyone to decide on their own answer. The correct answer was given once all teams had made their choices and a running total of the team scores announced before the next workshop activity began.

### Training, workshop development and content

Final year UG student projects are completed between October and May and workshops were delivered between January and April. The overall organisation of this eight month project, including important events and objectives, is given in Table 1. Within one week of meeting the chosen students and providing them with a detailed overview of the project, an ‘elevator pitch’ session was timetabled in which each student stated the subject of their workshop and several workshop activity ideas (the term ‘elevator pitch’ refers to the notion that the description should be short enough to convey the key aspects within the time it takes for an elevator ride: no more than a few minutes). This enabled the academic team to assess key attributes such as public speaking skills, confidence, sociality and creativity and identify talents as well as areas for which extra support or training might be provided. Prospective workshop dates were suggested and students were asked to prepare short summaries of their workshops and a title to aid the recruitment of schools, which also began at this time.

**Table 1:** Project timetable.

Month	Events
October	Ethical authorisation received; Group meeting: students receive first detailed overview of the project; Group meeting: elevator pitch session (discussion of initial ideas); Identification of available workshop dates; Schools receive information packs; Students submit two page summary of their workshop; Two further group meetings; One-on-one meeting with each student to elaborate ideas
November	Interested schools receive consent forms (head teacher and parent); Student safeguarding seminar; Formal workshop design seminar; Obtain resources for workshops; Group meeting: discuss resources required; Group meeting: background to the research question/dissertation; One-on-one meeting with each student
December	Perform health and safety assessment of all workshops; Material prepared for introductory session; Produce workshop quiz questions; Produce in-workshop MCQs; Dress rehearsal
January	Student exam period; Submission of dissertation introduction; Workshop delivery
February	Workshop delivery
March	Workshop delivery
April	Workshop delivery; Students receive summary of results; Data analysis; Presentation at British Neuroscience Association Conference
May	Submission of dissertation; De-brief meeting; Student evaluation

A number of one-on-one meetings (with the lead academic) and group meetings were scheduled over the next three months in which ideas, workshop activities and strategies to tailor neuroscientific information to a young age group were developed. We found that discussions held as an entire group, facilitated by the lead academic, best aided the development of workshop ideas and content. This enabled each student to retain overall responsibility for their own workshop, the theme chosen, planning, delivery, and writing the dissertation yet, at early stages of the project, benefit from the support and creative input from the group as a whole.

### Assessment and evaluation

The primary dependent variable was the score from a pen-and-paper neuroscience quiz which was completed at the end of each workshop (day 0) and again one week later (day 7; extra quiz sheets and stamped, addressed envelopes were given to each school on the day of the workshop to allow these to be returned free of charge). Name and gender was indicated on the quiz sheet but data were anonymised during input. Ten minutes were allotted at the end of the workshop for completion of the quiz and the evaluation (printed on the reverse of the quiz sheet; see below). The quiz comprised eight MCQs that were devised jointly by the academics and students: four questions were unique to a given workshop and four were the same for all workshops. For instance, in a workshop entitled ‘Inside the Brain of Usain’, on the topic of exercise and the autonomic nervous system that used athletes such as Usain Bolt as the central theme, two questions were ‘Nerves make muscles [blank]’ (with four possible answers: Contract, Expand, Stretch or Lengthen); and ‘What do adrenal glands make?’ (Water, Adrenaline, Electricity or Urine). Questions that were used across all workshops included ‘What is the name of the outside of the brain?’ (Cortex, Thalamus, Hippocampus or Hypothalamus) and ‘What do nerve cells make to send information?’ (Steam power, Water, Electricity or Radio waves).

Secondary dependent variables were obtained from the results of an evaluation that was completed by the pupils immediately after the first neuroscience quiz (on day 0). The evaluation was presented on the reverse of the neuroscience quiz and, thus, were linked to the quiz scores. Questions in the evaluation were designed to find out if the material was engaging, appropriate to the pupils' age, whether they learned something new, if it was enjoyable and whether the pupils enjoyed the experience to the extent that they would like the group to deliver another similar workshop in the future. We also explored the elements of each workshop that pupils enjoyed most and least. The evaluation comprised five statements that pupils were required to indicate their level of agreement by way of a Likert scale (1 = disagree, 5 = agree) and two open questions in which answered could be written into text boxes. The five statements were: S1) The session was interesting, S2) The session was easy to understand, S3) I learned new information, S4) The session was fun, S5) I would like these people to come back again. The two open questions were ‘What did you like most about the workshops?’ and ‘What did you like least?’. Given the nature of the assessment and evaluation, a very small number of returns were ‘spoiled’ for reasons such as no attempt being made to answer the questions or the questions were not completed in the allotted time. The number of quizzes or evaluations that were discounted for such reasons are given in the results section where necessary. Further, more constructive, evaluation of the workshops was provided by the completion of evaluation forms by classroom teachers in order to identify to the academics where specific improvements that could be made to the project as a whole to enhance the experience for both school pupils and UG students.

### Data analysis and statistics

Both quiz score and evaluation feedback data were found to be non-normally distributed as determined by D'Agostino & Pearson omnibus normality tests and, therefore, non-parametric statistical tests were used throughout. Quiz score and feedback data were divided by gender and Kruskal-Wallis (non-parametric) one-way analysis of variance (ANOVA) tests performed with Dunn's multiple comparison tests performed where appropriate. To examine the effect of delivery style on score improvement, the difference between quiz scores collected on the day of the workshops and one week later were also analysed which, due to the necessity to match pupils, invariably contained fewer pupils than the original quiz. This reduced pupil number was due to, for example, missing names on quiz sheets or absences from school. All statistical analysis was performed in Prism 6 (Graphpad, San Diego, CA, USA); data are reported in means ±95% confidence intervals throughout.

## Results

### Workshop delivery style has no impact on short-term retention of information

**Figure 1:**
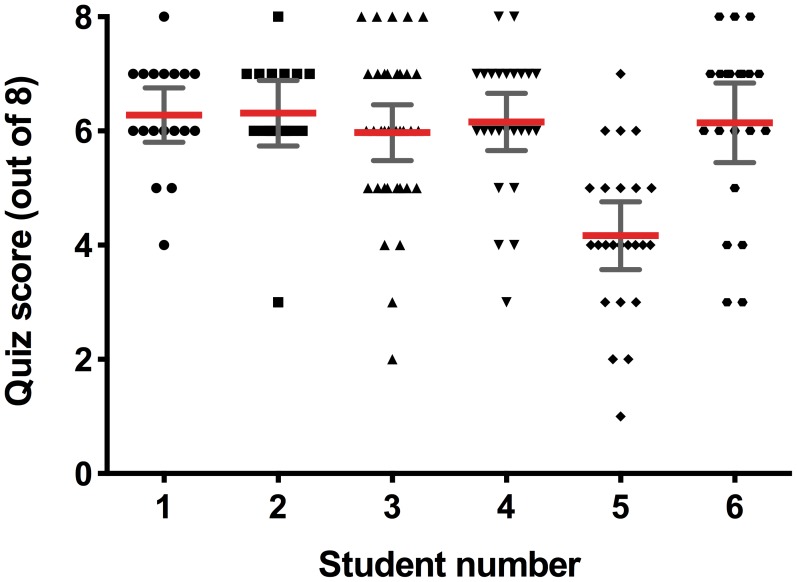
Individual pupil neuroscience quiz scores on the day of the workshop from six classes in the ‘No risk’ condition (each delivered by a different undergraduate student). Quiz scores in class #5 were significantly less than scores in all other classes (see text for explanation) and were, therefore, removed from the analysis (n = 139; Kruskal-Wallis followed by Dunn's test, *** p<0.001).

The immediate effect of workshop delivery style was assessed with a pen-and-paper neuroscience quiz performed at the end of each 90 minute workshop. In order to ensure that workshop quiz scores were comparable, Kruskal-Wallis one-way analysis of variance (ANOVA) tests were performed to compare quiz score data collected on the day of the workshop in each condition (i.e. comparing scores from each of the six student's classes). Only in the ‘No risk’ condition were quiz scores from one student's workshop (3.57±0.59) found to be significantly different from all other classes in that condition (student #5 in Figure 1; average of all other class scores = 6.14±0.24; Kruskal-Wallis statistic = 31.61, p<0.0001, number of values = 139; Dunn's test reported quiz score as different from all scores from all five other classes, mean rank differences = 43.32 to 53.33, p<0.001). This could be explained due to the quiz and feedback period at the end of this workshop being unexpectedly curtailed and, thus, these data were excluded (a total of 24 pupils).

**Figure 2:**
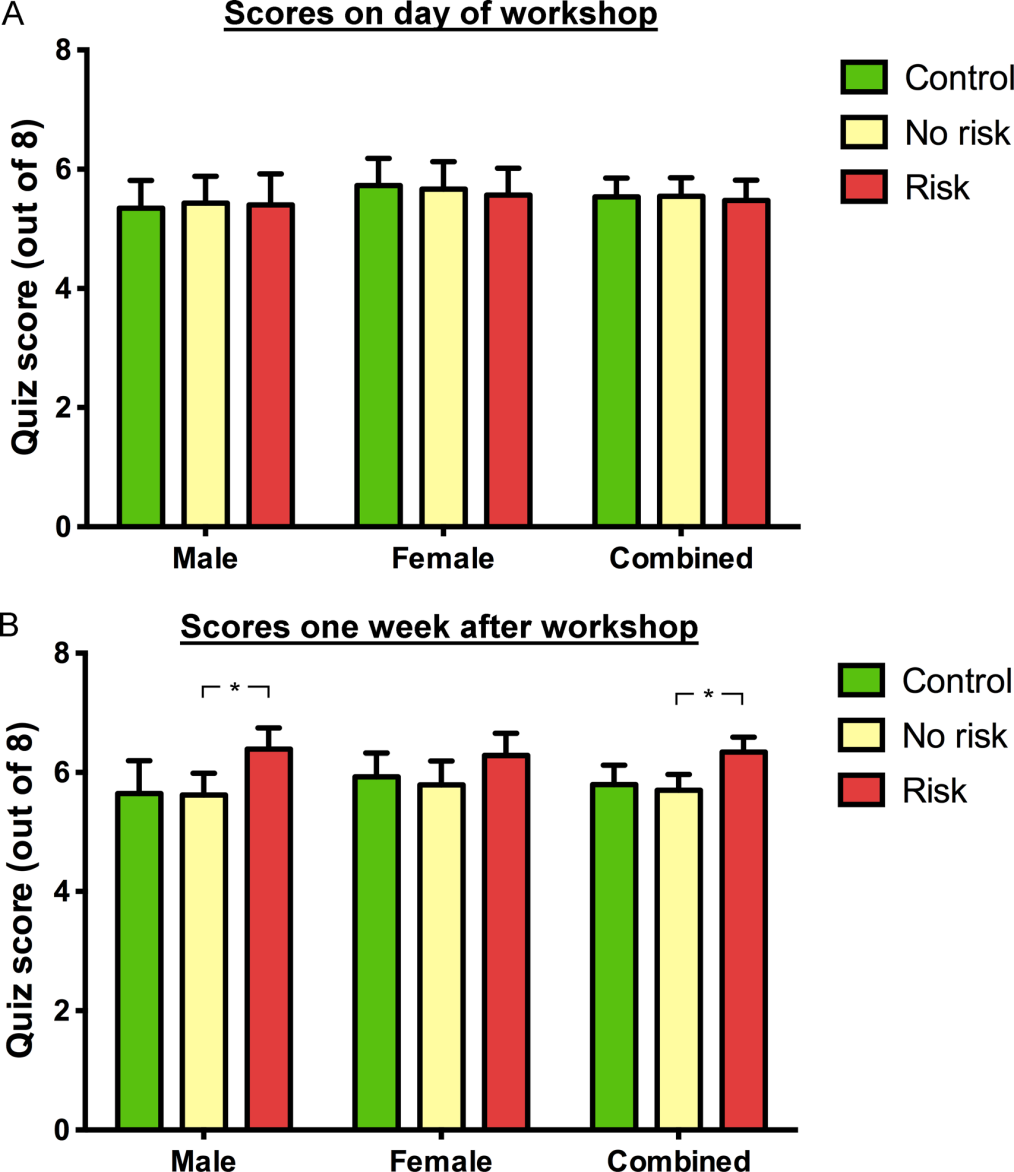
Average neuroscience quiz scores as overall scores and separated into gender. 2A: Scores on the day of the workshop. Total number of pupils in ‘Control’, ‘No risk’ and ‘Risk’ groups were 149, 139 and 154 respectively. 2B: Scores one week after the workshop. Total number of pupils in ‘Control’, ‘No risk’ and ‘Risk’ groups were 133, 131 and 141 respectively (Kruskal-Wallis followed by Dunn's test, ** p<0.01).

There was no difference in quiz scores compared between the three experimental conditions when all pupils were examined together (Figure 2A right-side; Kruskal-Wallis test statistic = 3.328 p = 0.189, total number of values = 418; spoiled quiz sheets = 6); average scores in the ‘Control’, ‘No risk’ and ‘Risk’ conditions were 5.81±0.26, 6.14±0.24 and 5.80±0.26, respectively. There was also no effect of workshop delivery style when pupils were divided into boys (Figure 2A left-side; Kruskal-Wallis test statistic = 2.296 p = 0.317, total number of values = 213; spoiled quiz sheets = 5), for which average scores in the ‘Control’, ‘No risk and ‘Risk’ conditions were 5.64±0.37, 6.03±0.39 and 5.78±0.39, respectively, or girls (Figure 2A centre; Kruskal-Wallis test statistic = 2.247 p = 0.325, total number of values = 205; spoiled quiz sheets = 1), for which average scores in the ‘Control’, ‘No risk’ and ‘Risk’ conditions were 5.99±0.36, 6.25±0.27 and 5.82±0.36, respectively.

### Longer-term retention of information is improved by using risk-based learning games

When follow-up quiz scores were collected one week after the neuroscience workshops, a significantly increased score was found for all pupils combined, and for boys specifically (Figure 2B). Average scores for all pupils in the ‘Control’, ‘No risk’ and ‘Risk’ conditions were 5.80±0.33, 5.70±0.27 and 6.30±0.26, respectively (Kruskal-Wallis test statistic = 11.590 p = <0.01, total number of values = 405; Dunn's test mean rank difference between Risk and No risk conditions = 46.84, p<0.01; spoiled quiz sheets = 43). Average scores for boys in the ‘Control’, ‘No risk’ and ‘Risk’ conditions were 5.65±0.55, 5.62±0.36 and 6.38±0.36, respectively (Kruskal-Wallis test statistic = 9.475 p = <0.01, total number of values = 204; Dunn's test mean rank difference between Risk and No risk conditions = 29.56, p<0.01; spoiled quiz sheets = 25). Average scores for girls in the ‘Control’, ‘No risk’ and ‘Risk’ conditions were 5.93±0.40, 5.79±0.40 and 6.28±0.37, respectively (Kruskal-Wallis test statistic = 3.712, total number of values = 201; spoiled quiz sheets = 18).

**Figure 3:**
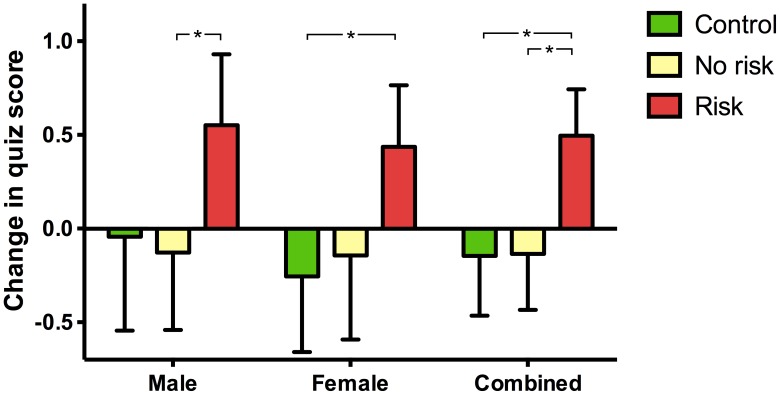
Change in quiz score between day 0 and day 7 on a pupil-by-pupil basis. Total number of pupils in ‘Control’, ‘No risk’ and ‘Risk’ groups were 89, 89 and 113 respectively (Kruskal-Wallis followed by Dunn’s test, * p<0.05, ** p<0.01).

To examine the change in individual pupils' quiz scores between the two test sessions, only pupils for which quiz scores could be identified at, and matched between, both time points were used. Classes at the very beginning of the project could not be matched due to names not being given on quiz sheets; further pupils had to be removed from analysis due to being absent at the second time point or failing to include their name. Quiz scores from a total of 291 pupils were successfully matched between the two time points (Control = 89, No risk = 89, Risk = 113); the difference in quiz scores between these times are shown in Figure 3. There was a significant effect of the ‘Risk’ condition over both ‘Control’ and ‘No risk’ conditions when all pupils were examined together (Kruskal-Wallis test statistic = 11.93, p<0.01, number of values = 291; mean rank difference between ‘Control’ vs. ‘Risk’ = 32.15, p<0.05; mean rank difference between ‘No risk’ vs. ‘Risk’ = 35.36, p<0.01). Compared to their performance on the day of the workshop, pupils in the ‘Risk’ condition scored 0.50±0.25 marks higher one week later; in the ‘Control’ and ‘No risk’ conditions, pupils scored −0.15±0.32 and −0.14±0.30 marks, respectively, compared to the first time they completed the quiz. For boys, scores in the ‘Control’, ‘No risk’ and ‘Risk’ conditions were −0.04±0.50, −0.13±0.41 and 0.55±0.38, respectively (Kruskal-Wallis test statistic = 6.62, p<0.05, number of values = 151; mean rank difference between ‘No risk’ vs. ‘Risk’ = 21.19, p<0.05). For girls, scores in the ‘Control’, ‘No risk’ and ‘Risk’ conditions were −0.26±0.40, −0.14±0.45 and 0.44±0.33, respectively (Kruskal-Wallis test statistic = 6.86, p<0.05, number of values = 140; mean rank difference between ‘Control’ vs. ‘Risk’ = 20.02, p<0.05).

### Pupils were more interested and believed they learned more in workshops that featured risk-based learning games

**Figure 4:**
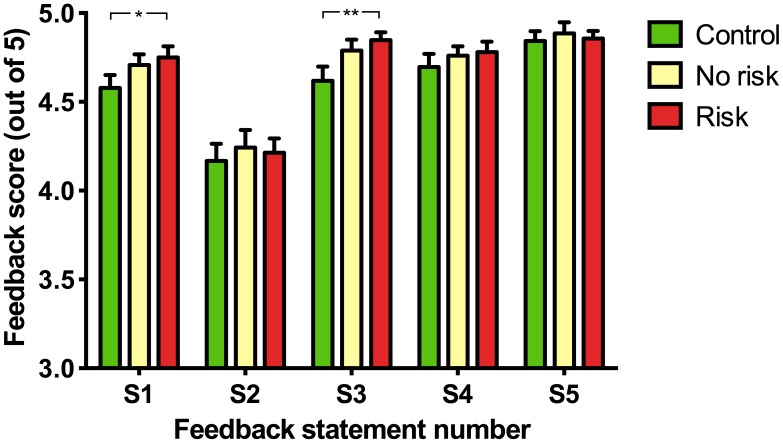
Pupil feedback. Pupils were asked to indicate their agreement to the following five statements by using a Likert scale (1 = strongly disagree to 5 = strongly agree): S1) The session was interesting, S2) The session was easy to understand, S3) I learned new information, S4) The session was fun, S5) I would like these people to come back again (Kruskal-Wallis followed by Dunn's test, * p<0.05, ** p<0.01).

Once pupils had answered all questions on the neuroscience quiz at the end of the workshop, a short evaluation was completed. This required each pupil to rate their agreement with five statements, S1–S5 (Figure 4). Average agreement ratings to all questions in all conditions was greater than 4 (agree). Pupils rated statements #1 (‘The session was interesting’) and #3 (‘I learned new information’) significantly higher in the ‘Risk’ than in the ‘Control’ group (S1: Kruskal-Wallis test statistic = 7.366 p = <0.05, total number of values = 328; Dunn's test mean rank difference between ‘Risk’ and ‘Control’ conditions = 24.22, p<0.05; S3: Kruskal-Wallis test statistic = 9.861 p = <0.01, total number of values = 328; Dunn's test mean rank difference between ‘Risk’ and ‘Control’ conditions = 23.77, p<0.01). Ratings for statements #2, #4 and #5 were not significantly different between the groups (S2: Kruskal-Wallis test statistic = 0.64, total number of values = 327; S4: Kruskal-Wallis test statistic = 2.64, total number of values = 328; S5: Kruskal-Wallis test statistic = 2.28, total number of values = 328).

### Teacher evaluation

Evaluations were only completed by teachers who had remained in the classroom during the workshops (n = 17) and were returned anonymously. The results of the quantitative feedback is shown in Table 2. Over 94% of teachers agreed that the workshops were well organised, contained age-appropriate information, were understandable and delivered clearly by enthusiastic presenters. The teachers believed that the pupils enjoyed the workshops and learned new information and would both recommend and be willing to participate in future workshops. In addition to ratings of the nine statements, teachers were given the option to provide qualitative information in the form of specific elements of the workshop they liked as well as suggestions for improvement. All teachers took the opportunity to provide (often multiple) positive comments. Of these, twelve highlighted the interactivity and breadth of experience the pupils could enjoy; seven focused on the communication skills (listening and clarity of speech) and enthusiasm of the students; five highlighted how engaging the workshops were, and three praised how a range of different learning styles were addressed by the activities. Three statements exemplify the range of comments provided: “The helpers kept the children engaged, they listened and spoke clearly so children understood well. There were a range of activities, a good mix of learning styles addressed. I learnt new things about the brain too!”, “The workshops were well-planned, brilliantly delivered, engaging and interactive without losing the academic content. Outstanding!” and “Our class love science and became even more enthusiastic when ‘real scientists’ with props/lab coats visited and gave the children more in-depth, exciting information and tasks.” Nine teachers provided suggestions for improvement. Of these, two merely expressed the desire to have more information on workshop tasks that the pupils enjoyed most; one comment indicated that some tasks required slightly more classroom space (school halls were requested but were occasionally unavailable). Three further comments concerned differentiation; for instance, one pupil in one workshop with special educational needs became disengaged, whilst some pupils chose answers to MCQs more quickly than others. One school in which two classes were included in the study indicated they would have liked a joint de-brief at the end of the workshops (prevented by the experimental design). Two comments regarded the initial difficulty of the risk-based game; students were placed with each individual class group to ensure that pupils understood the rules and what was expected of them.

**Table 2:** Statements were rated on a scale of 1–5, with 1 indicating ‘strongly agree’ and 5 indicating ‘strongly agree’.

Statement	Average rating (SD)	Agreement, %
I received sufficient information about the project beforehand	4.24 (0.83)	76.5
The workshops were well organised	4.65 (0.61)	94.1
Information was delivered at an appropriate level for the pupils	4.59 (0.51)	100
Delivery of instructions was clear and understandable	4.53 (0.62)	94.1
The presenters were enthusiastic	4.94 (0.24)	100
The pupils enjoyed the workshops	4.88 (0.33)	100
The pupils learned new information from the workshops	4.88 (0.33)	100
I would recommend such workshops to other schools	4.82 (0.39)	100
I would be willing to participate in future outreach projects	4.82 (0.53)	94.1

Agreement was judged by an answer of 4 or above. Number of teachers = 17.

## Discussion

The current study utilised UG student-led science workshops in local schools to investigate the impact of risk-based learning games on retention of information. Workshop feedback showed that pupils found the ‘Risk’ workshops more interesting than ‘No risk’ or ‘Control’ workshops and the involvement of risk also resulted in significantly higher recall of workshop information by pupils one week later. This result will be discussed by describing what is known about the effects of risky environments on brain function and also consider how the use of external rewards to incentivise participation in the learning game may also influence academic performance.

The potential of risk-based learning games to improve learning has been repeatedly highlighted and studies have been performed on the effects of such games upon, for example, galvanic skin responses of participants, how fair the games are and levels of uncertainty that participants prefer [9], [28]. Puzzlingly, the question of whether or not risk-based learning games can actually improve learning was never examined and, to the best of our knowledge, has never before been examined in a controlled manner. Risky play during infancy is believed to offer an important mechanism through which children overcome fears [29] and may still offer much to older children and adults by increasing arousal levels, especially when choices are made about how much risk to endure in a game [30]. The impact of risk-based learning games on the retention of information in the current study was measured by scores on a neuroscience quiz taken at two time points: immediately following science workshops and one week later. Although there were no differences between quiz scores in the ‘Risk’, ‘No risk’ and ‘Control’ conditions at day 0 (Figure 2A), scores from pupils in the ‘Risk’ group one week later were significantly higher (Figure 2B). When the change in quiz scores between the two time points was examined on a pupil-by-pupil basis, the average change in quiz score from pupils in the risk condition were higher than both non-risk and control conditions (Figure 3). In studies that assess retention of information over time, the actual measure in practice is the amount of information that has been forgotten. However, in the current study, scores in the ‘Risk’ condition one week after the workshop were higher than on the day of the workshop. In other words, knowledge *increased* in the week following the workshop. A clue as to why overall knowledge increased in one group comes from the pupil feedback data. More pupils in the risk workshops found their workshops interesting and believed they learnt new information (Figure 4). Although the quiz scores showed objectively that they didn't learn more than other pupils, the fact that they found the risk-involving workshops more interesting may have resulted in discussion and the sharing of workshop material outside of the classroom, thereby increasing the overall scores one week later. Thus, an indirect effect of risk-based learning games may have predominated over more direct effects of the learning game on memory consolidation and later recall.

Involvement in the risk-based game in all ‘Risk’ workshops was incentivised by the promise of a ‘brain-related prize’ for the winning team, which was only revealed at the end of the workshop. The use of external rewards in education is controversial but common [31], [32] and it is not yet certain whether rewards can motivate pupils to work harder or undermine intrinsic motivation by encouraging pupils to focus too much on the reward itself [33], [34]. It is likely that outcomes are dependent on both the particular paradigm utilised and the circumstances in which rewards are offered [33] but individual differences in emotional reactions to a given reward are also a vitally important variable and can ultimately determine whether a piece of information is either remembered or forgotten [35]. The presence of reward in the current study may have contributed to the increase in quiz score one week after the workshop by reminding the class of the workshop and prompting discussion of what was learned; the use of rewards to incentivise participation in the risk-based learning game is therefore one limitation of the current study. This raises the question whether a non-tangible reward would have had the same effect; current studies in our laboratory are hoping to shed light onto this by examining learning reinforcement strategies such as how tangible rewards or praise differentially influence pupil engagement and learning. A further limitation of this study is the necessary use of pupils' self-report for measures such as levels of interest in, and their belief that they learned new information from, the workshops. Such measures were not the primary focus of the study however and used as supporting evidence to probe the differences between workshop style on the more reliable and quantitative assessment of pupil information retention.

The cognitive and emotional states of learners during learning has, unsurprisingly, been shown to influence later recall (e.g. [36]) and, in response to calls for greater evidence-based practices in education [37], [38], [39], [40], the fledgling field of educational neuroscience is attempting to understand the conditions that are optimal for learning to take place and to extricate the different factors that contribute to an effective learning environment [41], [42], [43], [44], [45]. Regardless of the mechanisms of action, the investigation into particular learning games that exploit one or more neural processes in the developing brain, or are effective simply because they introduce novelty to a classroom, may constitute a fruitful avenue of educational neuroscience research if explored using appropriately controlled experimental designs such as the one utilised here.

## Conclusions

We have shown that the inclusion of risk-based learning games is an effective means by which pupils can be engaged and retention of information improved. Whilst a focus of future studies will be to elucidate the precise mechanisms by which the learning games promote retention, we have demonstrated a practical approach to evaluate topical questions in educational neuroscience and contribute to the growing evidence base in education. In so doing, we have also validated and fully described a new model that combines PE, undergraduate science communication training and classroom research.
